# A Mature-Tomato Detection Algorithm Using Machine Learning and Color Analysis †

**DOI:** 10.3390/s19092023

**Published:** 2019-04-30

**Authors:** Guoxu Liu, Shuyi Mao, Jae Ho Kim

**Affiliations:** 1 Computer Software Institute, Weifang University of Science and Technology, Shouguang 262-700, China; 201693257lgx@pusan.ac.kr; 2 Department of Electronics Engineering, Pusan National University, Busan 46241, Korea; msy0725@pusan.ac.kr

**Keywords:** tomato detection, harvesting robots, machine learning, color analysis

## Abstract

An algorithm was proposed for automatic tomato detection in regular color images to reduce the influence of illumination and occlusion. In this method, the Histograms of Oriented Gradients (HOG) descriptor was used to train a Support Vector Machine (SVM) classifier. A coarse-to-fine scanning method was developed to detect tomatoes, followed by a proposed False Color Removal (FCR) method to remove the false-positive detections. Non-Maximum Suppression (NMS) was used to merge the overlapped results. Compared with other methods, the proposed algorithm showed substantial improvement in tomato detection. The results of tomato detection in the test images showed that the recall, precision, and F_1_ score of the proposed method were 90.00%, 94.41 and 92.15%, respectively.

## 1. Introduction

Intelligent agriculture has attracted more and more attention around the world. Fruit harvesting robots are being rapidly developed due to their enormous potential efficiency. The first critical step for harvesting robots is detecting fruits autonomously. However, it is very difficult to develop a vision system that is as intelligent as humans for fruit detection. There are many reasons, such as uneven illumination, nonstructural fields, occlusion, and other unpredictable factors [1].

Intensive efforts have been made in vision system research for harvesting robots. Bulanon et al. [2] proposed a color-based segmentation method for apple recognition using the luminance and red color difference in the YCbCr model. Mao et al. [3] used the Drg-Drb color index to segment apples from their surroundings. The L*a*b* color space was employed to extract ripe tomatoes [4]. These methods use only color features for fruit detection and heavily rely on the effectiveness of the color space used. However, it is difficult to select the best color model for color image segmentation in real cases [5]. Furthermore, relying only on color features causes the loss of much of the other visual information in the image, which was proven to be very efficient for object recognition [6].

Kurtulmus et al. [7] proposed a green citrus detection method for use in natural outdoor conditions by combining Circular Gabor Texture features and Eigen Fruit. They reported a 75.3% accuracy. This method uses several fixed thresholds for detection. A method using feature image fusion was utilized for tomato recognition [8]. The a*-component image from the L*a*b* color space and the I-component image from the YIQ color space were fused by wavelet transformation, and then an algorithm based on an adaptive threshold was used to implement the detection.

Researchers have attempted to use various of sensors for fruit detection to overcome the problems of illumination variation and occlusion [9,10,11,12]. To locate cherries on a tree, Tanigaki et al. [10] used red and infrared laser scanning sensors, which prevented the influence of sunlight. Thermal and visible images were fused to improve the detection of oranges by Bulanon et al. [11]. Xiang et al. [12] employed a binocular stereo vision system for tomato recognition, and 87.9% of the tomatoes were recognized correctly. These techniques usually provided better results than conventional methods based on RGB color image. This is mainly due to the fact that similar reflectances in the visible light frequency band may show different results in the non-visible band. Nevertheless, the high cost of the sensors makes such methods difficult to commercialize.

More and more researchers are using machine learning in computer vision tasks, including fruit detection [1]. Ji et al. [13] proposed a classification algorithm based on an SVM for apple recognition, and the success rate of recognition reached 89%. An AdaBoost ensemble classifier was combined with Haar-like features and employed for tomato detection in greenhouse scenes [14]. A color analysis method was used to reduce false detections. Tomato fruits were detected using image analysis and decision tree models, and 80% of the tomatoes were detected [15]. Kurtulmus et al. [16] conducted comparison experiments for peach detection in natural illumination with different classifiers including several statistical classifiers, a neural network, and an SVM classifier, which were combined with three image scanning methods. An SVM classifier and a bag-of-words model were used for pepper detection [17].

The Histograms of Oriented Gradients (HOG) descriptor was proposed for pedestrian detection [18]. The HOG features behaved better than other features in detecting pedestrians. Motivated by the HOG features and machine learning methods, the goal of this study is to develop an approach to detect mature tomatoes in regular color images by combining an SVM classifier [19] and the HOG features. This study extends previous work [20]. Firstly, all the datasets are preprocessed through an illumination enhancement method. Then, the HOG features extracted from the training sets are used to train the SVM. In the detection stage, a coarse-to-fine scanning method is proposed to detect tomatoes in the entire image with different resolutions. Next, a False Color Removal (FCR) method is used to eliminate the false positive results. Finally, the Non-Maximum Suppression (NMS) method is applied to merge the overlapped detections.

The remainder of this paper is organized as follows. Section 2 presents the theoretical background. Section 3 describes the proposed tomato detection methods. Section 4 discusses the experimental results, and Section 5 presents the conclusions.

## 2. Theoretical Background

### 2.1. Histograms of Oriented Gradients Feature Extraction

Dalal and Triggs first proposed using HOG [18] as features for pedestrian detection. Due to its efficiency in pedestrian detection, the HOG feature has been widely used since then. The HOG can capture the shape information of an object and is invariant to geometric and photometric transformations. It can also deal with slight occlusion. However, to our knowledge, there is little research on fruit detection using HOG. Thus, in this work, HOG features are used in tomato detection.

**Figure 1:**
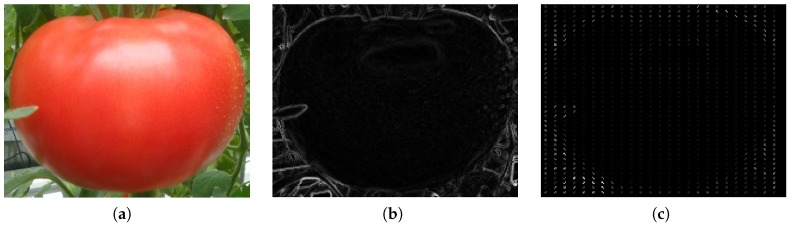
An example of Histograms of Oriented Gradients (HOG) descriptors: (**a**) An original sample, (**b**) the magnitude of the gradient image, and (**c**) a visualization of the HOG descriptors.

HOG is a descriptor that encodes the shape of an object. It divides an image into a number of cells. For each cell, a 1-D histogram of gradient directions or edge orientations over each pixel in the cell is calculated. All the histogram entries are combined to form the representation of the image. For a better illumination invariance, a local response contrast-normalization method is employed, which is performed by accumulating a measure of the local histogram energy over a block and normalizing all the cells of the block with the results. Figure 1 shows an example of HOG features of a tomato.

### 2.2. Linear SVM

**Figure 2:**
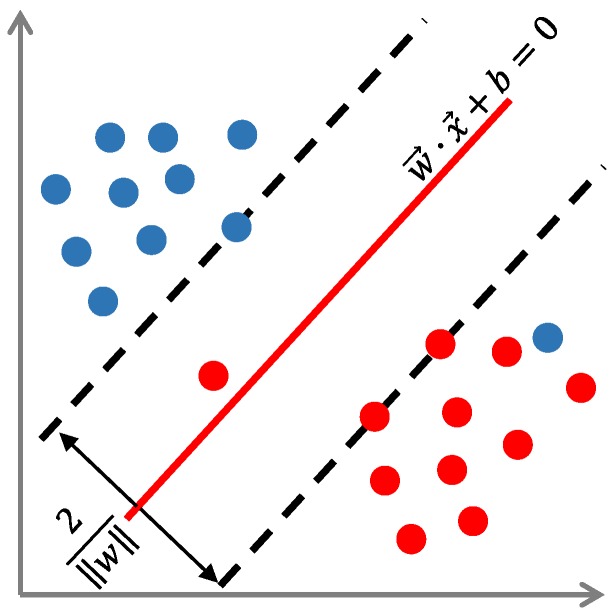
A linear Support Vector Machine (SVM) case.

The principle of linear SVM [19] is to find a hyperplane that can maximize the distance from the support vectors to the hyperplane. In Figure 2, the equation $\vec{w}\cdot \vec{x}+b=0$ denotes the separating hyperplane. The two positive samples (red) and one negative sample (blue) which are on the margins are called support vectors. The support vectors determine the separating hyperplane. In some cases, there are some outliers that cannot be separated linearly. In these cases, a slack variable $\epsilon_{i}$ is introduced to deal with the outlier data while accepting a reasonable error. The decision function $f\left(x\right)=sign\left(\vec{w}\cdot \vec{x}+b\right)$ is solved using Equation (1):

(1) $$\begin{array}{c} \min_{\alpha }\;\frac{1}{2}\sum_{i=1}^{N}\sum_{i=1}^{N}\alpha_{i}\alpha_{j}y_{i}y_{j}\left(\vec{x_{i}}\cdot \vec{x_{j}}\right)-\sum_{i=1}^{N}\alpha_{i}\\ s.t.\;\;\sum_{i=1}^{N}\alpha_{i}y_{i}=0\\ \;\;\;0\leq \alpha_{i}\leq C,\;i=1,2,\cdots ,N \end{array}$$

where $\alpha_{i}$ and $\alpha_{j}$ are the Lagrange multipliers and $\vec{x_{i}}$ and $y_{i}$ are the feature vector and label of sample *i*. C is the penalty parameter, and N is the total number of samples.

### 2.3. The Non-Maximum Suppression for Merging Results

The NMS is a method for reducing repetitive detections or for merging the nearby detections around one object. It has been widely used [21,22] and proved efficient in object detection. It relies on the classification probability from the classifier and the overlap area among the bounding boxes to merge the results. After detection using the proposed method, there may be multiple detections pointing to the same tomato. Thus, we adopt NMS as a post-processing step to address this problem.

## 3. Materials and Methods

### 3.1. Image Acquisition and Preprocessing

**Figure 3:**
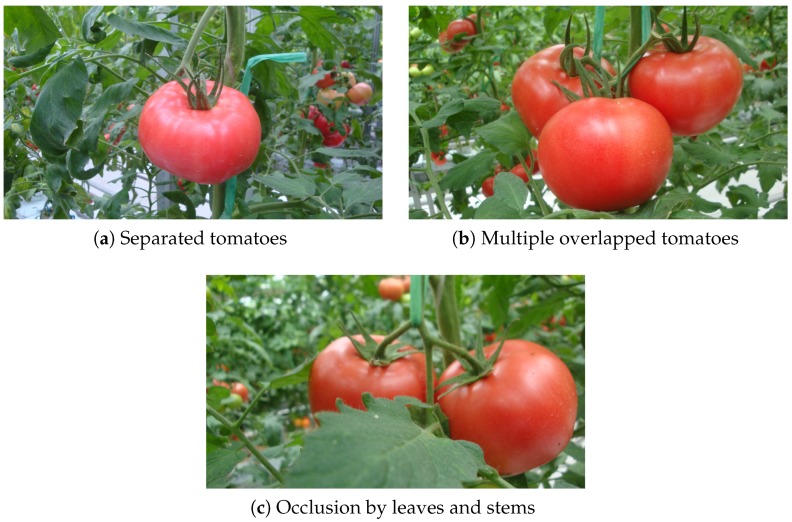
Images of tomatoes in different conditions: (**a**) separated tomatoes, (**b**) multiple overlapped tomatoes, and (**c**) occlusion by leaves and stems.

To develop and evaluate the proposed algorithm, images of tomatoes in a greenhouse were acquired in late December 2017 and April 2019 in Vegetable High-tech Demonstration Park, Shouguang, China. A total of 247 images were captured using a color digital camera (Sony DSC-W170) with a resolution of 3648 × 2056 pixels. The photographs were taken at distances of 500–1000 mm, which is in accordance with the best operation distance for the harvesting robot. As shown in Figure 3, the growing circumstances of the tomatoes vary and include separated tomatoes; multiple overlapped tomatoes; and tomatoes occulted by leaves, stems, or other non-tomato objects. To speed up the image processing, all of the images were resized to 360 × 202 pixels using a bicubic interpolation algorithm. The dataset has been made publicly available [23].

**Figure 4:**
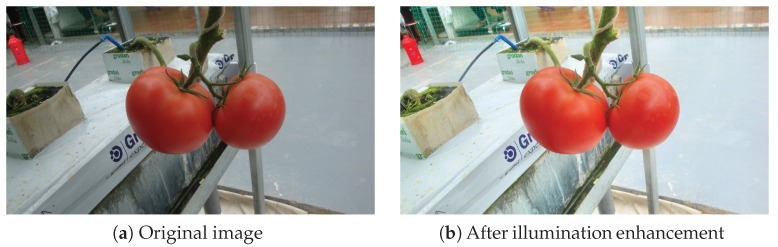
An example of illumination enhancement: (**a**) before enhancement and (**b**) after enhancement.

An illumination enhancement method was used to decrease the effect of uneven illuminations. The image was first converted from RGB space to Hue-Saturation-Intensity (HSI) space. The I layer was then split, and a natural logarithm function was applied to each pixel. Next, the Contrast Limited Adaptive Histogram Equalization (CLAHE) method [24] was applied to the transformed I component. Finally, the H, S, and processed I layers were combined to obtain the final enhanced image. This procedure was performed on all the images as a preprocessing step before training the classifier. An example of image enhancement is shown in Figure 4.

### 3.2. The Dataset

**Figure 5:**
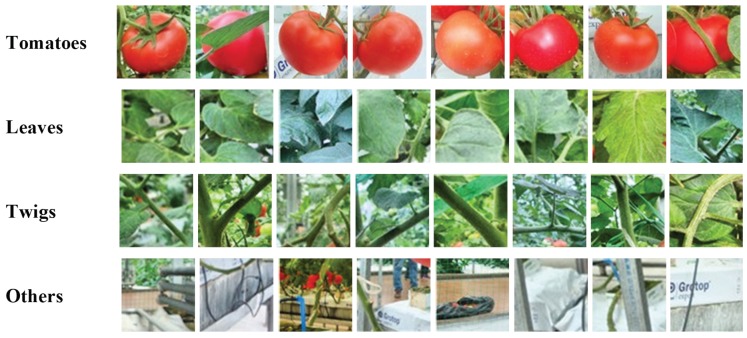
Examples from the dataset. Top row: tomato samples; lower row: background including leaves, stems, and other objects.

A total of 247 images were used for the experiment. To train the SVM classifier, 100 images were randomly selected from the captured images, 72 images were used for validation set, and the remaining 75 images were used for the test. From the training images, 207 tomato samples and 621 background samples were manually cropped to construct a training set. The training samples were augmented with random rotations of 0°–360°. This doubles the size of the training set (1656 samples in all). All of the cropped samples were resized to 64 × 64 pixels to unify the size. The tomato samples contained a margin of about 5 pixels on all the sides. The background samples were randomly cropped to contain leaves, stems, strings, and other objects, and all the samples were separately labeled, 1 for the tomatoes and −1 for the backgrounds. Some examples for the datasets are shown in Figure 5.

### 3.3. Overview of the Detection Algorithm

**Figure 6:**
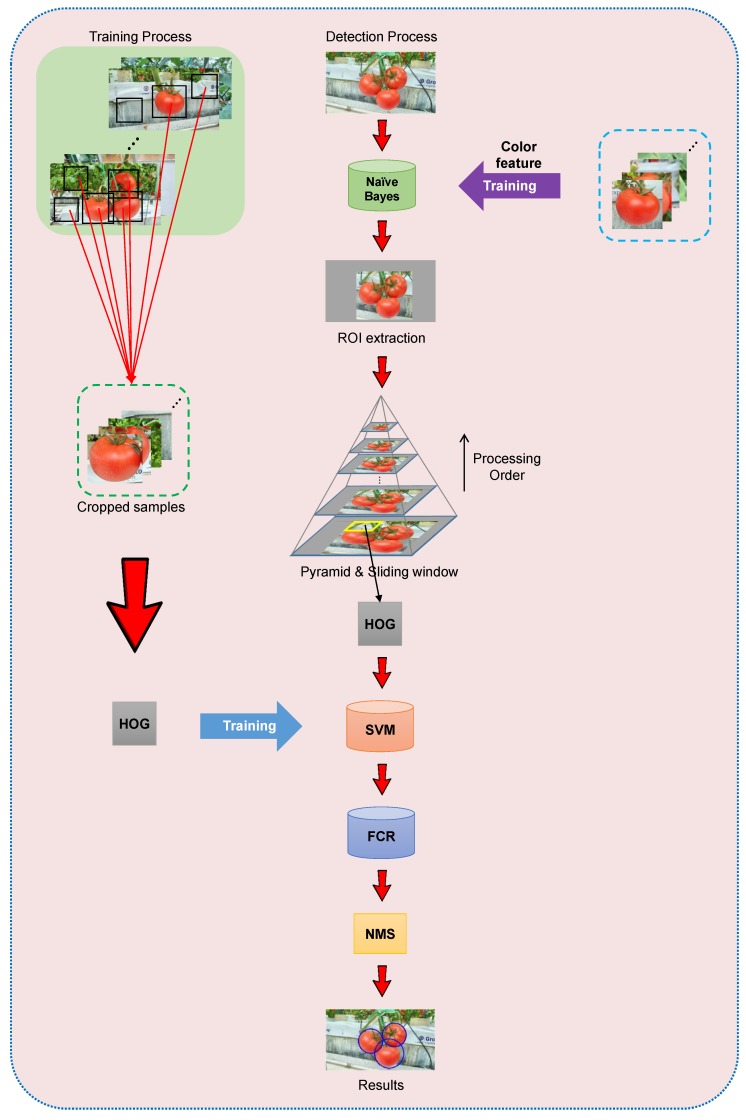
A systematic view of the proposed method.

**Figure 7:**
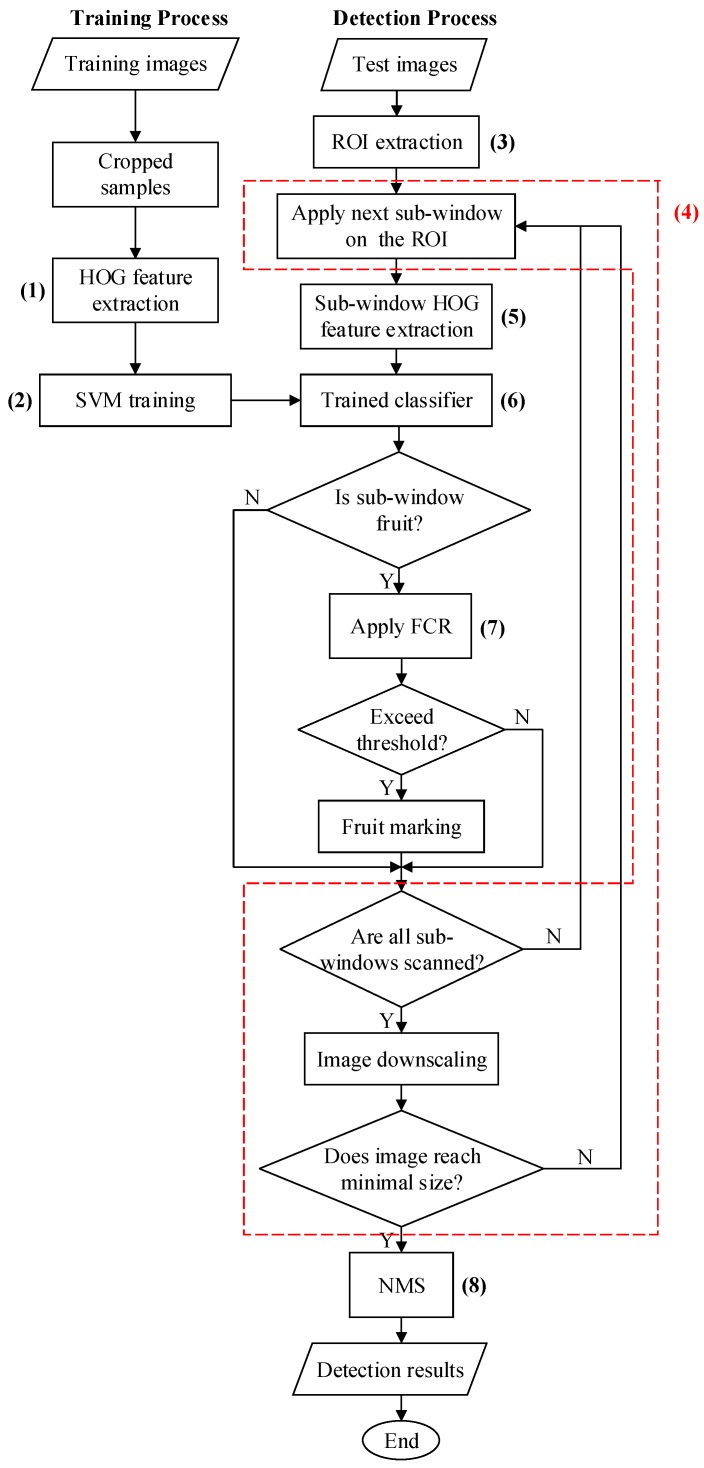
A flowchart of the developed algorithm: The numbers correspond to the steps described previously.

Figure 6 and Figure 7 show a systematic view and flowchart of the developed algorithm. The process can be summarized in the following steps:

(1) Extracting the HOG features of the training samples
(2) Training an SVM classifier using the extracted features and corresponding labels
(3) Extracting the Region-of-Interest (ROI) on the test image using a pretrained Naive Bayes classifier
(4) Sliding a sub-window on the ROI of the image with different resolutions using an image pyramid
(5) Extracting the HOG features of each sub-window
(6) Recognizing tomatoes within the pretrained classifier
(7) Performing FCR to remove any false positive detections
(8) Merging the detection results using the NMS method

### 3.4. Image Scanning Method

After training the SVM classifier using the training set, a coarse-to-fine detection framework is used to detect tomatoes. The pseudo code and detailed detection process are described in Algorithm 1.

All the pixels are classified as belonging to tomatoes or the background using a Naïve Bayes classifier (NB) trained on color features. Since mature tomatoes are red, three color transformations are performed to distinguish the fruits from background: R−G, R−B, and R/(R+G+B). After classification, a binary image is obtained, in which white pixels represent the potential tomatoes and black pixels represent the potential background.

**Algorithm 1:** The pseudo code of the scanning method.

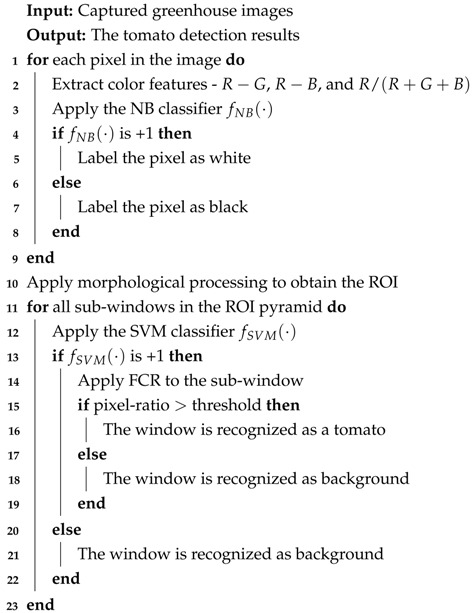



**Figure 8:**
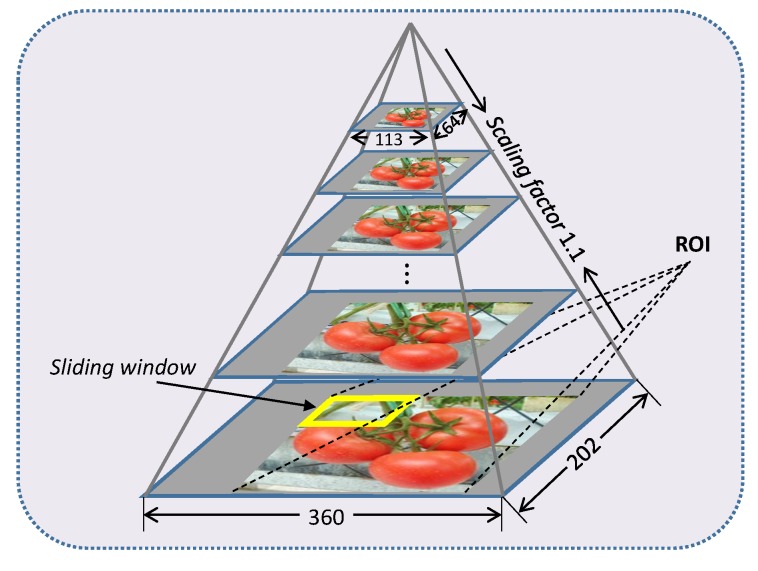
A sketch map of the sliding window and image pyramid.

Next, a morphological processing is applied to the binary image, and the Region-of-Interest (ROI) is extracted. A sliding window is applied to the ROI and slides with a fixed step. At each step, the sub-window is input to the pretrained SVM classifier to be classified as a tomato or not a tomato. If the sub-window is classified as a tomato, then FCR is used to implement further classification. After the sliding window slides all over the ROI, the image is downscaled by a fixed scaling factor, followed by the same sliding process until a defined minimum size is reached. The sliding window size is 64×64 based on the size of the tomatoes in the images. The sliding step and minimum size of the scaled image are set to 16 and 113×64, respectively. The image scaling factor is 1.1, which downscales the image by 10% at each step. A sketch map of the sliding window and image pyramid is shown in Figure 8.

### 3.5. False Color Removal

All sub-windows of the image could be classified using the SVM classifier. However, there are some false positive detections after the classification, and a false positive elimination method is needed to reduce them. Color features play an important role in fruit detection, especially when the fruits have a different color from the background. A False Color Removal (FCR) method is proposed for false detection elimination. The sub-window image is binarized using a color feature which is derived as shown below, and then, the ratio of the number of white pixels to the number of all pixels in the sub-window is calculated. If the ratio exceeds a threshold of 0.3, the sub-window is classified as a tomato. Otherwise, it is classified as the background.

The cost function minimization [19] was applied as follows to obtain the color feature for binarization. A total of 897 samples including tomatoes and the background were chosen as the training set. The R, G, and B components of the RGB color model were extracted, and the mean value of each component over all the pixels of each sample was calculated to represent the sample. The tomato samples were labeled as 1, and the background samples were labeled as −1. Motivated by Cortes [19], a separating plane in Equation (2) is needed to separate tomatoes and background in the R-G-B coordinates:

(2) $$\begin{array}{c} \vec{w}\cdot \vec{x}+b=0 \end{array}$$

where $\vec{x}$ is the feature vector (R,G,B). $\vec{w}$ and *b* are the weight vector and bias of the separating plane, respectively.

It is derived by minimizing the cost function *L* in Equation (3):

(3) $$\begin{array}{c} \min_{\vec{w},b,\epsilon }\;L\left(\vec{w},b,\epsilon \right)=\frac{1}{2}{∥\vec{w}∥}^{2}+\sum_{i=1}^{M}\epsilon_{i}\\ s.t.\;\;y_{i}\left(\vec{w}\cdot \vec{x_{i}}+b\right)\geq 1-\epsilon_{i},\;i=1,2,\cdots ,M\\ \;\;\;\epsilon_{i}\geq 0,\;i=1,2,\cdots ,M \end{array}$$

where $\vec{x_{i}}$ and $y_{i}$ are the feature vector $\left(R_{i},G_{i},B_{i}\right)$ and label of the sample *i*, respectively. *M* is the number of samples, and $\epsilon_{i}$ is the slack variable of sample *i*, which is used to deal with the outliers.

The color feature derived for sub-window binarization is 0.16×R−0.093×G−0.037×B−11.032, and the threshold is 0.

### 3.6. Experimental Setup

In this study, all experiments of the developed algorithm were performed on Python version 3.5 with an Intel^®^ Core^TM^ i5-4590 CPU@3.30 GHz. Several experiments were conducted to validate the performance of the developed method. The datasets used in the experiments are listed in Table 1. Some examples of the results in each step are shown in Section 4.2, Section 4.3, Section 4.4 and Section 4.5. Three indexes were used to evaluate the performance of the proposed algorithm and recently developed algorithms: recall, precision, and F_1_ score, which are defined by Equations (4)–(6):

(4) $$Recall=\frac{Correctly\;identified\;tomato\;count}{Total\;number\;of\;tomatoes}\times 100\%$$

(5) $$Precision=\frac{Correctly\;identified\;tomato\;count}{Total\;number\;of\;detections}\times 100\%$$

(6) $$F_{1}=\frac{2\times Precision\times Recall}{Precision+Recall}\times 100\%$$

## 4. Results and Discussion

### 4.1. Results of Different HOG Features

HOG features with different cell sizes, block sizes, and number of orientation bins were tested on the validation sample set. The Receiver Operating Characteristic (ROC) curve and the Area Under the Curve (AUC) [25] were used to evaluate their performance. In this section, the default HOG has the following characteristics: 8 × 16 pixel blocks of four 4 × 8 pixel cells; a linear gradient voting into 10 orientation bins in 0°–180°; and an L2 block normalization.

**Figure 9:**
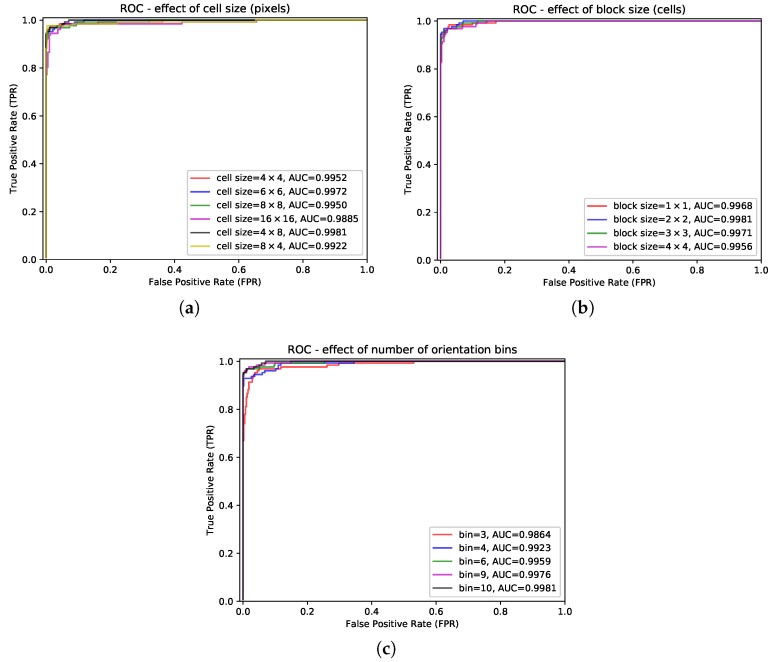
HOG feature performances with different specifications: (**a**) the effect of cell size (pixels), (**b**) the effect of block size (cells), and (**c**) the effect of the number of orientation bins.

The cell size was tested with 4 × 4, 8 × 8, 16 × 16, 4 × 8, and 8 × 4 pixels. The ROC curves are shown in Figure 9a. The HOG feature with 4 × 8 pixel cells gave the highest AUC.

The block size was tested with 1 × 1, 2 × 2, 3 × 3, and 4 × 4 cells. Figure 9b shows that the HOG feature with 2 × 2 cell blocks got the best result.

The number of orientation bins was tested with 3, 4, 6, 9, and 10. As shown in Figure 9c, the HOG feature with 10 orientation bins achieved the best performance.

In this study, the HOG feature with 4 × 8 pixel cells, 2 × 2 cell blocks, and 10 orientation bins was used for experiments.

### 4.2. Results of the Image Scanning Method

**Figure 10:**
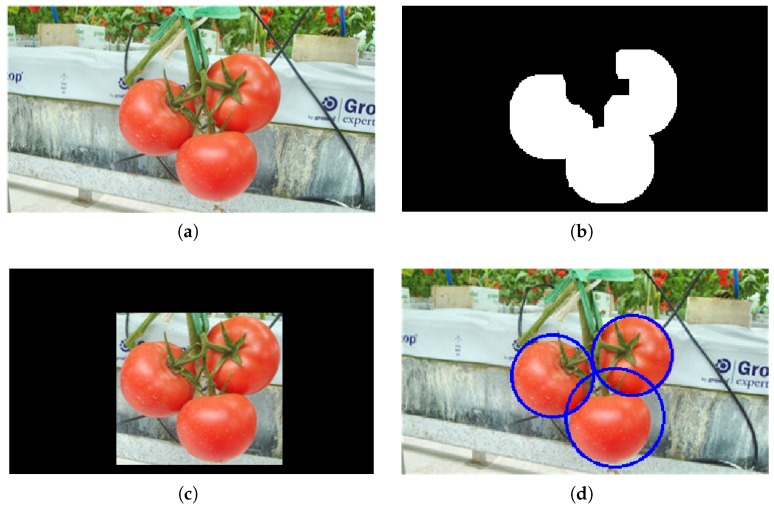
An example of scanning method 2: (**a**) an original image, (**b**) the results after NB classification and the morphological process, (**c**) the extracted region of interest (ROI), and **(d)** the detection results.

An example of the coarse-to-fine framework proposed for tomato detection is shown in Figure 10. After classification by the NB classifier and the morphological operation, the binary image in Figure 10b was obtained, and the ROI extracted is shown in Figure 10c. After ROI extraction, the area was reduced by more than 50%, which means the method can accelerate the detection speed by about two times. The ROI still contained the detection targets. Finally, the detection was performed on only the ROI, and the final results are shown in Figure 10d.

### 4.3. Results of the SVM Classifier

**Figure 11:**
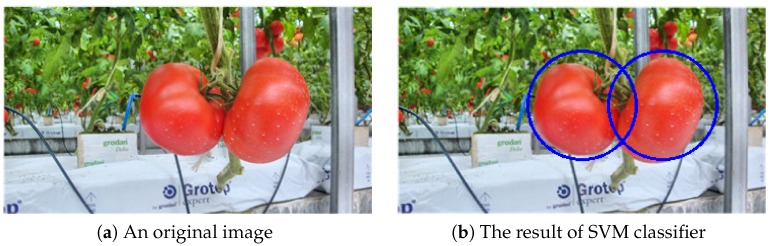
An example of SVM classification: (**a**) before and (**b**) after applying the SVM classifier.

The SVM uses a linear kernel with a penalty parameter C=1. It is implemented using the open-source scikit-learn package [26]. Examples from before and after applying the SVM classifier are shown in Figure 11. Both the tomatoes were correctly detected with the inscribed circle of the bounding box.

### 4.4. Results of False Color Removal (FCR)

**Figure 12:**
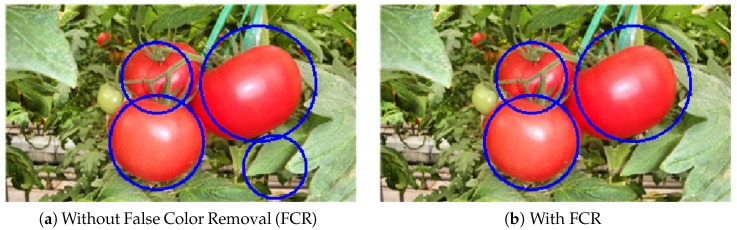
The results of the FCR: (**a**) without FCR and (**b**) with FCR.

After detection using the SVM classifier, the tomatoes can be found along with some false positives (i.e., the backgrounds). Thus, the proposed FCR method is then applied to reduce the false positives. Examples from before and after applying FCR are shown in Figure 12. A generated false positive is shown in Figure 12a since the shape of the region is similar to a circle. It was successfully removed after performing FCR, as shown in Figure 12b.

### 4.5. Results of the Non-Maximum Suppression

After detection using the SVM classifier and the FCR in each sub-window, there are many sub-windows classified as tomatoes, and some of them correspond to the same one. Therefore, the NMS method is introduced.

**Figure 13:**
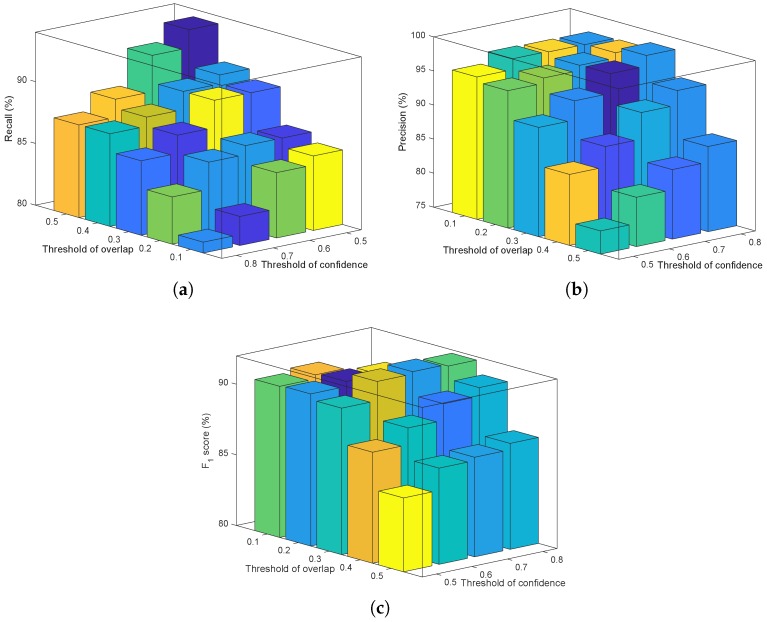
The effect of different overlap and confidence thresholds on (**a**) recall, (**b**) precision, and (**c**) the F_1_ score.

The performance of the NMS mainly depends on choice of overlap and confidence thresholds. The overlap–confidence threshold combinations were tuned on the validation detection set. The impacts of the thresholds on recall, precision, and F_1_ score are shown in Figure 13. The thresholds of 0.3 (overlap) and 0.7 (confidence), which could get the best F_1_ score, were selected as the optimal thresholds and used in this paper.

**Figure 14:**
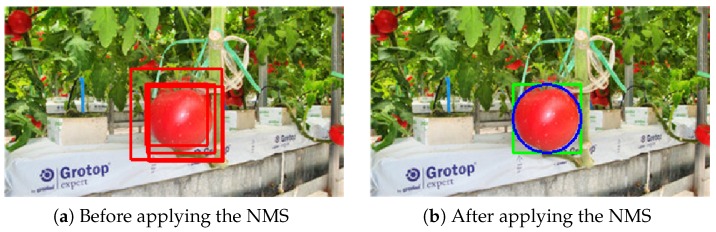
An example of the NMS: (**a**) before and (**b**) after applying the NMS.

An example of using the NMS is shown in Figure 14. The bounding box that had the highest prediction probability was chosen for the final prediction compared to other boxes which overlapped with it over the threshold of 0.3.

### 4.6. Performance of the Developed Classifier for Cropped Samples

Manually cropped tomato samples were used in an experiment to evaluate the proposed method. Both the training and test sets were utilized, and the results are shown in Table 2. The recall and precision on the test set were 96.85% and 98.40%, respectively, which shows that the proposed method is effective for tomato detection.

### 4.7. Robustness of the Proposed Algorithm to Illumination

**Figure 15:**
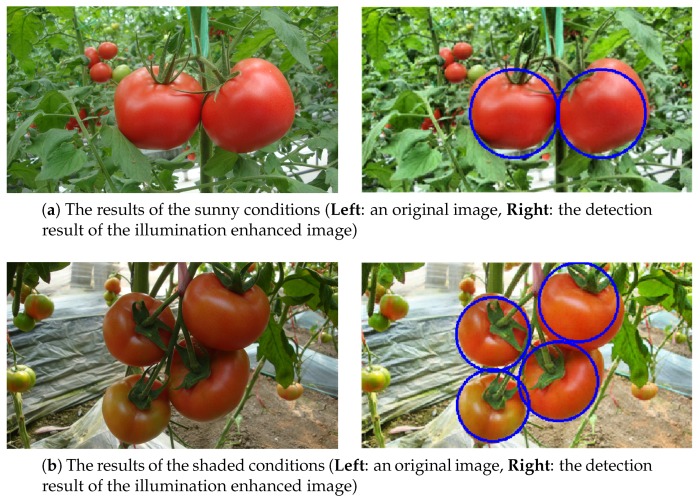
An example of the detection results under different lighting conditions: (**a**) sunny conditions and (**b**) shaded conditions.

The performance of the proposed method was evaluated using 75 tomatoes in sunny conditions and 75 in shaded conditions. The results are shown in Table 3. For the sunny conditions, a 90.67% accuracy was achieved, while an 89.33% accuracy was obtained for the shaded conditions. The false positive rates were 5.56% and 5.63% for the sunny and shaded conditions, respectively. The results were comparable which proved that the proposed method was insensitive to illumination variation inside the greenhouse environment. This was mainly due to two factors: the illumination enhancement in the preprocessing step and the illumination normalization in the HOG–feature calculation process. Some examples of the results are shown in Figure 15.

### 4.8. Performance of the Proposed Method under Separated, Overlapped, and Occluded Conditions

The tomatoes under separated, overlapped, and occluded conditions were also tested. For the overlapped conditions, the tomatoes overlapped with each other in the image, while the occluded conditions referred to the tomatoes being blocked by leaves or stems. Notably, some of the overlap or occluded areas reached over 50%, which maked the detection task much more challenging. The detection results under each condition are shown in Table 4, and 135 tomatoes were detected out of the total of 150 tomatoes. The overall correct identification rate was 90.00%.

**Figure 16:**
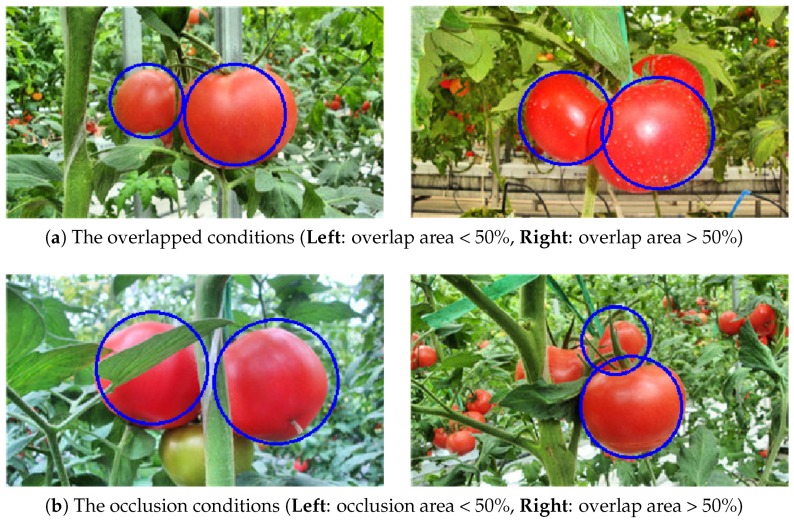
The detection results under different conditions: (**a**) the overlapped conditions and (**b**) the occlusion conditions.

All the tomatoes were correctly identified under the separated conditions as expected. For the overlapped and occluded conditions, the results became worse. Under overlapped conditions, the correct identification rate was 91.14% due to high overlap areas between tomatoes in some cases reaching over 50%. When the overlap area was under 50%, the proposed method could detect most of the tomatoes, but it failed if the area exceeded 50%. An example in Figure 16a illustrates this phenomenon. In Figure 16a (left), two overlapped tomatoes were both detected, while in Figure 16a (right), just two tomatoes were correctly detected and not the top-right one, which was largely covered by another tomato.

A similar explanation accounts for the results of the occluded conditions. As a result, 42 tomatoes were correctly detected out of the 50 tomatoes, and the missed ones were mostly due to heavy occlusion by leaves or stems. The correct identification rate was 84.00%. An example is shown in Figure 16b. In Figure 16b (left), two tomatoes that are blocked by leaves and stems were still correctly detected. However, in Figure 16b (right), only two tomatoes were detected, while the one in the left that was largely occluded by leaves and stems was not detected.

**Figure 17:**
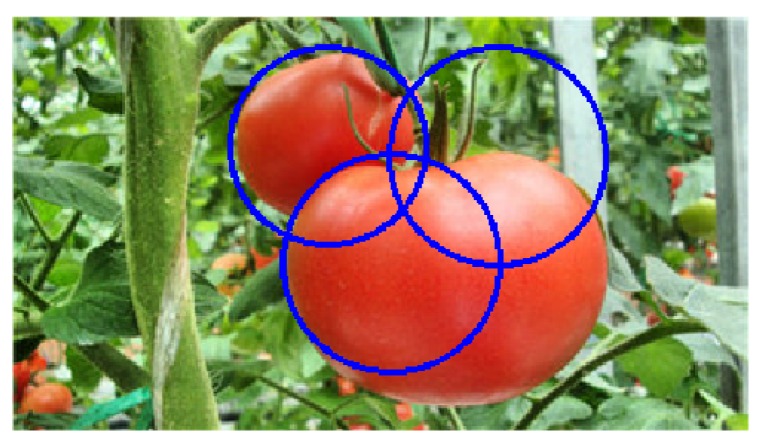
A false positive example.

In addition, there were some false positives in the detection results, which was mainly due to the various tomato sizes. When several detections corresponded to the same tomato, only one was considered to be the true positive, and the others were all regarded as false positives. An example is shown in Figure 17.

### 4.9. Comparison with Other Methods

Two other recently proposed methods [7,14] were compared with the proposed method. The first method [7] uses a Circular Gabor Filter and Eigen Fruit as features, and the other method uses an AdaBoost classifier [14], which uses a Haar-like feature as input. Moreover, one of the popular deep learning frameworks—YOLO (You Only Look Once) [27]—was also applied to evaluate its performance. Another experiment was set up using all of the same steps as the proposed method except for the false detection elimination step to test the effectiveness of the FCR. Table 5 shows the results. The proposed method achieved the second highest recall and had the second highest precision. This benefit from the better representation of the descriptor, the scanning framework, and the merging method.

The deep learning methods usually perform better than traditional methods in the face of big data. However, when the data is small or insufficient, they may be underfit due to the deep network structure, and the performance may be equal or even inferior to traditional methods. Table 5 shows the results.

The precision of the proposed method improved substantially after the FCR, which was largely due to the false positive elimination. To provide a more objective assessment, the F_1_ score [28] was calculated, which combined both the recall and precision together. Table 5 shows that the proposed method had the highest F_1_ score, which demonstrated that the method was effective and could be applied for the detection of mature tomatoes.

## 5. Conclusions and Future Work

An algorithm was proposed to overcome the difficulties that harvesting robots face in fruit detection. The method used color images captured by a regular color camera. Compared with single-feature detection methods, the proposed method used a combination of features for fruit detection, including shape, texture, and color information. This approach can reduce the influence of illumination and occlusion factors. HOG descriptors were adopted in this work. An SVM classifier was used to implement the classification task. In the scanning stage, a coarse-to-fine framework was applied, and then, an FCR method was used to eliminate the false positives. Lastly, NMS was used to obtain the final results.

Several experiments were conducted to evaluate the efficiency of the proposed method. A total of 510 samples were used to validate the classification efficiency of the SVM classifier. The recall was 96.85%, and the precision was 98.40%. The results showed that the classifier with only HOG features can distinguish tomatoes from backgrounds very well. When it comes to detection, the correct identification rate is 90.67% in sunny conditions and 89.33% in shaded conditions. Similar results showed that the proposed method could reduce the influence of various illumination levels in the greenhouse environment. The correct identification rate was 100% for separated tomatoes, 91.14% for overlapped tomatoes, and 84.00% for occluded tomatoes, and a reasonable false positive rate was maintained. The missed tomatoes were mainly due to the area largely being blocked by other tomatoes or the background by over 50%. If the blocked area was less than 50%, most of the tomatoes could be detected correctly. Compared with other methods, the proposed method gave the best results. As a reference, the average processing time of one image was about 0.95 s.

However, there are still some problems in the proposed method. The accuracy is not satisfactory for the overlapped and occluded tomatoes, especially when the blocked area exceeds 50%. Another limitation is that the experiment was carried out in the harvesting stage. Therefore, most of the tomatoes of the experiment were ripen well and fully red. The authors believe that the detection of tomatoes at other stages including green and breaking red is also needed for the harvesting robot. Our future research will focus on further improving the detection accuracy and extension to other stages of tomatoes. Transfer learning [29,30] can also be applied with an extension of the datasets in the future.

## Figures and Tables

**Table 1:** The datasets for the experiments.

Dataset		Set Size
Training sample set		Tomato (414), Background (1242)
Validation sample set		Tomato (127), Background (383)
Validation detection set		Tomato (136)
Test detection set	Sunny	Tomato (75)
	Shadow	Tomato (75)
Test detection set	Separated	Tomato (21)
	Overlapped	Tomato (79)
	Occlusion	Tomato (50)

**Table 2:** The performance of the SVM classifier.

Set	Actual Categories	Samples Number	Classified Categories		Recall (%)	Precision (%)
			Tomato	Background		
Train	Tomato	414	414	0	100	100
	Background	1242	0	1242		
Validation	Tomato	127	123	4	96.85	98.40
	Background	383	2	381		

**Table 3:** The detection results of the proposed method under different lighting conditions.

Conditions	Tomato Count	Correctly Identified		Falsely Identified		Missed	
		Amount	Rate (%)	Amount	Rate (%)	Amount	Rate (%)
Sunny	75	68	90.67	4	5.56	7	9.33
Shadow	75	67	89.33	4	5.63	8	10.67
All	150	135	90.00	8	5.59	15	10.00

**Table 4:** The detection results of the proposed method under different conditions.

Conditions	Tomato Count	Correctly Identified		Falsely Identified		Missed	
		Amount	Rate (%)	Amount	Rate (%)	Amount	Rate (%)
Separated	21	21	100	1	4.55	0	0
Overlapped	79	72	91.14	4	5.26	7	8.86
Occlusion	50	42	84.00	3	6.67	8	16.00
All	150	135	90.00	8	5.59	15	10.00

**Table 5:** A comparison of several tomato detection methods.

Methods	Recall (%)	Precision (%)	Missed (%)	F_1_ (%)
Proposed (no FCR)	91.33	86.16	8.67	88.67
Proposed	90.00	94.41	10.00	92.15
AdaBoost [14]	77.33	94.31	22.67	84.98
CGF & EF [7] *	78.67	97.52	21.33	87.08
YOLO [27]	88.00	92.31	12.00	90.10

* CGF & EF refers to Circular Gabor Filter and Eigen Fruit.

## Abbreviations

The following abbreviations are used in this manuscript:

SVM	Support Vector Machine
HOG	Histograms of Oriented Gradients
CLAHE	Contrast Limited Adaptive Histogram Equalization
NB	Naive Bayes
FCR	False Color Removal
NMS	Non-Maximum Suppression
ROC	Receiver Operating Characteristic
AUC	Area Under the Curve
